# Characterization of ANXA1 in chemotherapy resistance of head and neck squamous cell carcinoma: insights from artificial intelligence and integrative bioinformatics analysis

**DOI:** 10.3389/fcell.2026.1769105

**Published:** 2026-02-26

**Authors:** Ling Zheng, Haiyan Yang, Yan Cheng, Binxue Xia, Honghui Liu, Hong Xiong

**Affiliations:** 1 School of Nursing, Southwest Medical University, Luzhou, Sichuan, China; 2 Department of Respiratory and Critical Care Medicine, Affiliated Hospital of Southwest Medical University, Luzhou, Sichuan, China; 3 Emergency Medicine Department, Southwest Medical University Affiliated Hospital, Luzhou, Sichuan, China

**Keywords:** artificial intelligence, chemoresistance, head and neck squamous cell carcinoma, multi-omics, ANXA1

## Abstract

**Background:**

Head and neck squamous cell carcinoma (HNSCC) exhibits intensive chemoresistance (CR), leading to frequent recurrence and poor prognosis; however, actionable biomarkers and therapeutic options remain limited.

**Methods:**

By utilizing bulk profiles of HNSCC patients (TCGA-HNSCC cohort and GSE6631) from TCGA and GEO databases, we identified CR-associated DEGs via Limma and WGCNA frameworks. Importantly, LASSO–Cox regression was utilized for the construction of a predictive model and identification of the CR-associated hub gene in TCGA-HNSCC cohort. In addition, predictive model performance was validated in the HNSCC patient bulk profile (GSE65858). Furthermore, the molecular and immune characteristics of the hub gene were estimated at HNSCC patient bulk (TCGA-HNSCC cohort) and single-cell (GSE163872) levels, especially in artificial intelligence (AI)-empowered virtual cells. Specifically, AI-driven therapeutic framework (RefLector) and molecular docking were performed for the recognition of an optimal therapeutic framework for the treatment of HNSCC by targeting the hub gene. Finally, the cariogenic role of the hub gene was evaluated in an *in vitro* study.

**Results:**

CR-associated DEGs can guide the risk stratification of HNSCC patients. ANXA1 was identified as a downregulated, malignancy-distributed, prognostic, and druggable biomarker for HNSCC patients, which was also associated with HNSCC progression. BRD-K10482608 can be considered a potential therapeutic agent for the treatment of HNSCC.

**Conclusion:**

Our study highlighted the CR in risk stratification for HNSCC patients and ANXA1 in the pathogenesis of HNSCC, which can guide personalized and precision medicine for HNSCC patients.

## 1 Introduction

Head and neck squamous cell carcinoma (HNSCC) is a highly heterogeneous epithelial malignancy of the upper aerodigestive tract with increasing global incidence and mortality (Barsouk et al., 2023; Daste et al., 2024). Its evolution is shaped by site-specific and etiologic factors, such as tobacco, alcohol, and HPV, together with multilayered molecular derangements, including recurrent mutations in TP53, PIK3CA, FAT1, and NOTCH pathway genes, pervasive copy-number alterations, and epigenetic dysregulation (Solomon et al., 2018; Mei et al., 2020; Bicci et al., 2024). In addition, an immunosuppressive tumor microenvironment—dominated by cancer-associated fibroblasts, M2-polarized macrophages, regulatory T cells, and aberrant extracellular matrix—provides further durable therapeutic benefit (Kitamura et al., 2020). Chemoresistance (CR) remains the principal clinical barrier to platinum-based regimens, which is caused by coordinated mechanisms encompassing enhanced drug efflux and metabolism, augmented DNA damage response, epithelial–mesenchymal transition and stemness, metabolic reprogramming, epigenetic remodeling, and microenvironment-driven immune evasion (Zeng et al., 2019; Wilczyński et al., 2024).

**FIGURE 1:**
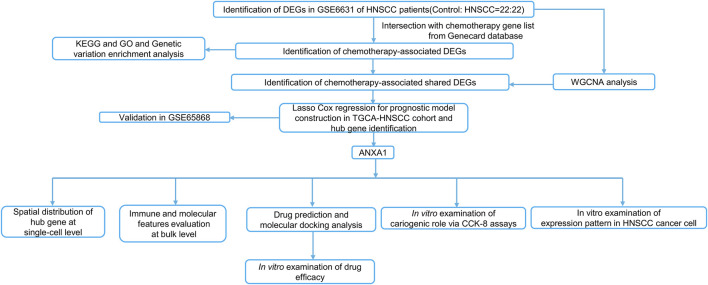
Workflow of this study.

To delineate CR-related biology and identify actionable targets for HNSCC patients, we implemented artificial intelligence (AI) and multi-omics across single-cell and bulk profiles across TCGA and GEO databases of HNSCC patients. We discovered that CR can guide the risk stratification for HNSCC patients and ANXA1 can be considered a CR-associated hub gene involved in HNSCC pathogenesis implicated by *in silico* and *in vitro* studies. In addition, an AI pipeline pointed out the potential therapeutic agents (BRD-K10482608) targeting ANXA1 for the treatment of HNSCC. Overall, our study provides additional insights into precision and personalized medicine for HNSCC patients via integrative AI and multi-omics pipelines. The workflow of this study is illustrated in Figure 1.

## 2 Methods

### 2.1 HNSCC bulk dataset acquisition

The GSE6631 (detected on GPL8300) gene expression dataset was retrieved from the GEO repository via the GEOquery package in R and included 22 normal mucosa tissue samples and 22 HNSCC mucosa tissue samples (Davis and Meltzer, 2007). The GSE65858 (detected on GPL10558) gene expression dataset was also retrieved from the GEO repository via the GEOquery package in R and included 270 HNSCC mucosa tissue samples (Davis and Meltzer, 2007). Both these datasets were subjected to normalization and standardization via the Limma package in R (Ritchie et al., 2015). The TCGA-HNSCC bulk profiles and the corresponding clinical information were acquired from TCGA database, and after transformation into TPM format, they were normalized and standardized via the edgeR package in R (Liu et al., 2021). We finally acquired 548 HNSCC mucosa tissue samples and 44 normal mucosa tissue samples. The list of chemotherapy resistance-associated genes was retrieved from the GeneCards database with the threshold greater than 1 (Xu S. et al., 2023). Histological slices of HNSCC patients and matched normal controls were acquired from The Human Protein Atlas database.

### 2.2 CR gene module identification via WGCNA analysis

In this work, the WGCNA package in R was utilized to build a gene co-expression framework to explore the relationships between gene expression patterns and clinical traits. Initially, genes with the lowest 50% median absolute deviation (MAD) were filtered out to reduce noise. Pairwise Pearson correlations were then computed and used, together with average linkage, to generate a weighted adjacency matrix (Langfelder and Horvath, 2008). A soft-thresholding power (β) was chosen to transform the correlations into an adjacency measure, which was further converted into a topological overlap matrix (TOM) (Langfelder and Horvath, 2008). Genes exhibiting similar expression behaviors were clustered into modules through hierarchical clustering based on TOM dissimilarity, setting a minimum module size of 50 (Langfelder and Horvath, 2008). Finally, module eigengene dissimilarities were calculated, an appropriate cut height was applied to the module dendrogram, and closely related modules were merged. To gain deeper insights into the biological significance of the candidate genes, we carried out a comprehensive functional enrichment analysis. The Gene Ontology (GO) framework—which encompasses three principal domains, namely, molecular function (MF), biological process (BP), and cellular component (CC)—was applied to systematically annotate gene functions. In parallel, pathway enrichment based on the Kyoto Encyclopedia of Genes and Genomes (KEGG) database was performed to elucidate high-level signaling networks and metabolic pathways associated with these targets. To achieve a more integrated understanding of the oncogenic mechanisms involving the selected mRNAs, the clusterProfiler package in R in accordance with the hallmark gene set downloaded from the MSigDB database was utilized to conduct GO term interrogation and detect KEGG pathways with significant enrichment (Yu et al., 2012).

### 2.3 CR prognostic signature construction and variable description

In TCGA-HNSCC cohort, we performed least absolute shrinkage and selection operator (LASSO) regression with 10-fold cross-validation, implemented in the glmnet package of R, for the construction of a CR-associated prognostic model and hub gene identification (Wang Q. et al., 2022). In addition, model performance was cross-estimated in TCGA-HNSCC cohort and the validation set (GSE65858) via KM, time-dependent RO, nomogram, and calibration analysis via survival, survminer, and ggplot2 packages of R. Subsequently, after acquisition of hub genes, we estimated the hub gene expression and prognostic performance in TCGA-HNSCC cohort and GEPIA database. Immune infiltration analysis was performed in retained TCGA-HNSCC dataset. To conduct a reliable immune score assessment, we used CIBERSORT algorithms to investigate the immune characteristics of hub genes in TCGA-HNSCC cohort (Chen et al., 2018). In addition, we also applied the ESTIMATE package in R to estimate the relationship between hub genes and immune and stromal scores in TCGA-HNSCC cohort (Wang et al., 2024). To investigate the functional relevance of candidate biomarkers, a single-gene GSEA was carried out using the clusterProfiler package in R in accordance with the hallmark gene set downloaded from the MSigDB database (Yu et al., 2012). For each target gene, its expression levels were correlated with all genes within each reference set, and the resulting coefficients were ranked to generate enrichment scores.

### 2.4 HNSCC single-cell dataset processing

Single-cell RNA sequencing (RNA-seq) data for GSE163872 (based on Illumina NovaSeq 6000, including 1 HNSCC patient tissue sample) were retrieved from the GEO repository. The processing of the single-cell RNA sequencing (scRNA-seq) data involved several key steps, including quality control (QC), dimensionality reduction, and the identification of markers, all of which were conducted using the Seurat R package (Tan et al., 2023). Quality control protocols were systematically implemented for each cell, adhering to the predetermined criteria that mandated gene counts ranging from 200 to 6,000, a unique molecular identifier (UMI) count surpassing 1,000, and a mitochondrial gene percentage below 10%. Following the quality control procedures, data normalization was conducted, facilitating the identification of 2,000 genes demonstrating significant variability for further exploration. Post-normalization, dimensionality reduction methods, particularly t-SNE and UMAP, were carried out. Cell-type annotations were performed using the scMayoMap algorithm available in R software (Yang et al., 2023). The expression profiles of the targeted genes were examined across the different annotated cell populations. The evaluation of copy-number variation (CNV) within individual cells was rigorously carried out utilizing the inferCNV R package, which is particularly relevant in studies focusing on epithelial cells to differentiate cancer cells (Chen et al., 2022). Intercellular communication networks were inferred through the use of the celltalker package in R software (Ma et al., 2024). Additionally, the energy metabolic pathways at the single-cell level among the annotated cell populations were analyzed using the scMetabolism package in R (Hartmann et al., 2021). ScTenifoldKnk was performed in this study to identify the regulation of hub genes in target cells through hub gene knockout (Osorio et al., 2022).

### 2.5 Drug enrichment and docking

The DrugReflector framework utilizes active learning techniques, utilizing transcriptomic data to pinpoint modulators associated with various disease phenotypes (DeMeo et al., 2025). Utilizing TCGA-HNSCC cohort, we implemented DrugReflector to identify the most effective therapeutic agents aimed at alleviating HNSCC. To evaluate the binding affinity of the optimal therapeutic agents with the hub gene, we conducted molecular docking studies. This molecular docking analysis was aimed at investigating the interactions between the selected drugs and the corresponding proteins (Wang Y. et al., 2022). The Protein Data Bank (PDB) files for the target protein (PDB ID: 1QLS) were obtained from the RCSB PDB, while the ligand SDF file (compound CID: 44616894) was sourced from the PubChem database. Following this, molecular docking was performed to estimate the binding affinities between the targeted proteins and the respective compounds. Initially, PyMOL software (version 2.6.0) was utilized to remove water molecules and ligands, retaining solely the protein backbone. Subsequently, the AutoDock Vina (version 4.2.6) tool was utilized to identify potential binding sites on the protein surface and to carry out flexible molecular docking (Wang Y. et al., 2022). This procedure involved calculating docking scores and binding affinities (Vina score, kcal/mol) for each identified binding site. The top five binding sites were ranked based on their binding energy, with the site exhibiting the lowest energy selected for further visualization in PyMOL. This visualization process elucidated the positions of hydrogen bonds associated with ligand binding as depicted in the resulting images. The findings were subsequently illustrated in PyMOL to represent the binding modes and hydrogen bonding interactions effectively.

### 2.6 Cell lines and culture

The HSC-3 (HNSCC) and AW-YCH191 (control) cell lines were procured from the Shanghai Academy of Biological Sciences, Shanghai, China, and were derived from human oral tissue. The HSC-3 and AW-YCH191 cell lines were maintained in Roswell Park Memorial Institute (RPMI) 1640 complete medium, enriched with a 1% antibiotic solution comprising penicillin–streptomycin and 10% fetal bovine serum (FBS, Gibco). In contrast, the HEK293T cell line, procured from the Shanghai Academy of Biological Sciences, Shanghai, China, was cultivated in Dulbecco’s modified Eagle medium (DMEM) supplemented with a 1% penicillin–streptomycin solution and 10% FBS. All procedures for cell passage were conducted under strictly controlled conditions (37 °C and 5% CO_2_) within a humidified incubator.

### 2.7 RNA extraction and qRT-PCR

Total RNA was extracted utilizing TRIzol reagent (TaKaRa, Beijing, China), followed by an assessment of its concentration, purity, and integrity through a NanoDrop spectrophotometer (Thermo Fisher Scientific, Waltham, MA, United States). For the reverse transcription step, 1 µg of total RNA was used in conjunction with HiScript II Q RT SuperMix for qPCR, which comprises both a gDNA wiper and a gDNA eraser (Vazyme, Shanghai, China). The concentration, purity, and integrity of the synthesized cDNA were subsequently evaluated using the aforementioned NanoDrop spectrophotometer. Quantitative reverse transcription polymerase chain reaction (qRT-PCR) was performed with SYBR Green MasterMix (11203ES50, Yeasen, Shanghai, China) and StepOne software version 2.3 (Applied Biosystems, Carlsbad, CA, United States) over 40 cycles, incorporating three biological replicates for each sample. Data analysis was carried out using the ΔΔCt (cycle threshold) method, with expression levels normalized to the reference gene *GAPDH*. The primer sequences utilized in the qRT-PCR experiments are detailed below:

ANXA1:

F 5′‐ACT​GCT​TCT​ACA​GGA​TTT​ATG​GTT‐3′;

R 5′‐CAA​AAA​GCA​GCC​CCC​ATC​AC‐3′.

GAPDH:

F 5′‐ GAG​AAG​GCT​GGG​GCT​CAT​TT‐3′;

R 5′‐ ATG​ACG​AAC​ATG​GGG​GCA​TC‐3′.

### 2.8 Western blotting

Following the administration of diverse treatments, the cells were thoroughly washed with ice-cold phosphate-buffered saline (PBS) (HyClone, Seattle, WA, United States) and subsequently collected through gentle scraping. Total protein extraction was accomplished by lysing the cells with radioimmunoprecipitation assay (RIPA) lysis buffer (Beyotime, Shanghai, China), supplemented with a combination of phosphatase inhibitors (Beyotime, China) and protease inhibitors (Beyotime, China). The resulting cell lysates underwent centrifugation at 14,000 × g for 15 min at 4 °C. Post-centrifugation, the lysates were denatured for 10 min in a 5× SDS-PAGE loading buffer (Beyotime, China). The proteins were then separated using SDS-PAGE and transferred to polyvinylidene fluoride (PVDF) membranes (Beyotime, China) for Western blot analysis. The membranes were blocked with NCM Blot blocking buffer (NCM Biotech, Suzhou, China) for 10 min. Following this, they were incubated with primary antibodies for 8 h at 4 °C and diluted in 5% bovine serum albumin (BSA) (Solarbio, Beijing, China). After incubation, the membranes were treated with secondary antibodies (Thermo Fisher Scientific, Waltham, MA, United States) and diluted in WB secondary antibody diluent solution (Beyotime, Shanghai, China) at a 1:1,000 dilution for 2 h at room temperature. Protein detection was carried out using an enhanced chemiluminescence (ECL) substrate (Thermo Fisher Scientific, Waltham, MA, United States). The quantification of protein expression was performed by analyzing the band densities of the target proteins using ImageJ software version 1.57, utilizing density values relative to the GAPDH protein. The primary antibodies used in this analysis included ANXA1 (ab214486, Abcam, United States: 1:1,000) and GAPDH (ab181602, Abcam, United States: 1:2,000).

### 2.9 Silencing via shRNA

The sequences designated for the silencing of ANXA1 were as follows:

shRNA targeting ANXA1: GCC​TTG​TAT​GAA​GCA​GGA​GAA.

These sequences were incorporated into the pLKO.1 lentiviral plasmid vector. Specifically, the plasmids that encode the shRNA were co-transfected with the VSV-G envelope plasmid and the psPAX packaging plasmid into HEK293T cells. The transfection was performed using Lipofectamine 2000 (Thermo Fisher Scientific), adhering to the manufacturer’s instructions. The growth medium was replaced the following day, and the lentivirus-containing supernatants were harvested 3 days after transfection, filtered, and subsequently used to infect the target cells in the presence of 4 μg/mL polybrene (Sigma-Aldrich). The HSC-3 cell line was then cultured at a density of 5 × 10^4^ cells per well in 24-well plates until 50%–70% confluency was reached. Following this, the standard culture medium was replaced with a diluted sh-ANXA1 lentiviral stock solution for transfection purposes. After 72-h incubation for 72 h, the cells underwent trypsinization and were washed with phosphate-buffered saline (PBS). The cells were then seeded into 10-cm culture dishes at a density of 500 cells per dish, and puromycin (Thermo Fisher Scientific) selection was conducted over a period of 3 weeks. The surviving clones were identified by the formation of cloning rings, after which they were expanded and subcloned using the limiting dilution technique.

### 2.10 Cell proliferation assays

Cells in the logarithmic growth phase underwent trypsinization, followed by enumeration and subsequent plating into 96-well plates at a density of 3,000 cells per well (n = 6). After incubation at specified time points (24, 48, 72, and 96 h), 10 µL of CCK-8 reagent was added to each well, and the plates were incubated for an additional 2 h. Absorbance at 450 nm was measured using a microplate reader. The cell proliferation rate was calculated using the formula (A_d − A_blank_d)/(A_4 h − A_blank_4 h). All experiments were performed in triplicate to ensure the reliability of the results. For IC_50_ validation, cells were plated in 96-well plates at a density of 2,000 cells per well (100 µL per well) and cultured for 24 h (at 37 °C and 5% CO_2_) after drug treatment. Following this, 10 µL of Cell Counting Kit-8 (C0038, Beyotime) reagent was added to each well, ensuring that bubble formation was minimized. The plates were then incubated for an additional 2 h. Absorbance readings at 450 nm were obtained using a microplate reader (EnSight, PerkinElmer, United States), and the cell survival rate was calculated in accordance with the manufacturer’s instructions.

### 2.11 Statistical analysis

In the context of bioinformatics analysis, all statistical assessments were performed using R software (version 4.2.2). The Wilcoxon test was utilized to evaluate the variances in the proportions of immune-infiltrating cells within the tumor microenvironment. Pearson correlation analysis was used to explore the interrelationships among various variables. A p-value or false discovery rate (FDR) of less than 0.05 was regarded as statistically significant. The data are expressed as mean ± standard deviation (SD), with significance levels indicated as *p < 0.05, **p < 0.01, and ***p < 0.001. In the experimental component, all statistical analyses were carried out using GraphPad Prism (version 8.0.2), ensuring that each experiment included a minimum of three biological replicates. The results are presented as mean ± SD. To assess differences between the two datasets, statistical analysis was conducted using either two-way ANOVA or Student's t-test, with a p-value of less than 0.05 considered statistically significant.

## 3 Results

### 3.1 Identification of CR-DEGs in HNSCC patients

**FIGURE 2:**
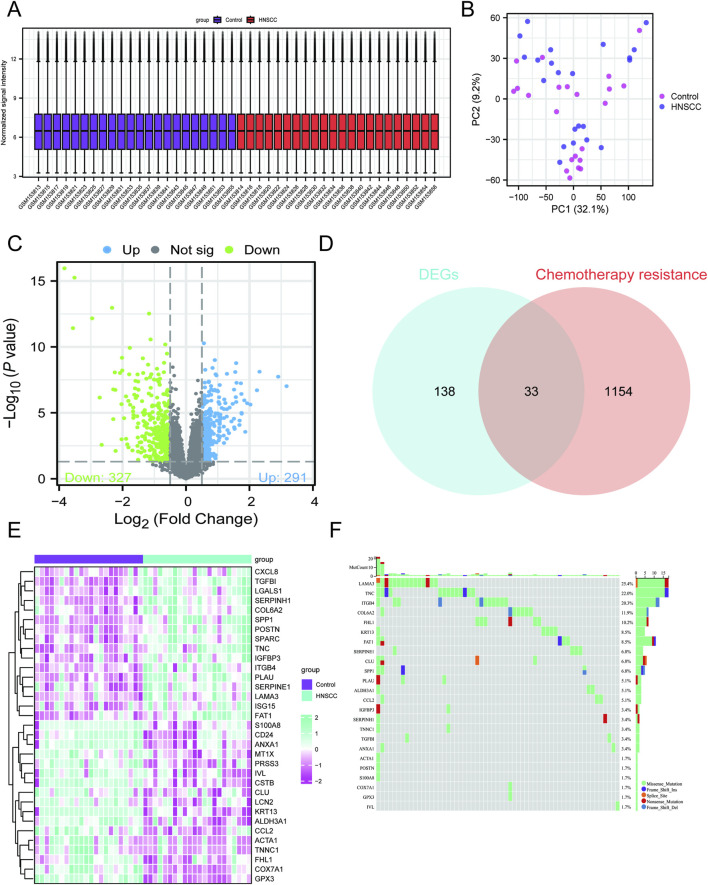
Identification of CR-DEGs in HNSCC patients. **(A)** Box plot of GSE243359. **(B)** PCA plot of GSE243359. **(C)** Volcano plot of DEGs in GSE243359. **(D)** Venn diagram of CR-DEGs. **(E)** Heatmap of CR-DEGs. **(F)** Diagram illustrating somatic mutations in the CR-DEGs. Data with a threshold of p < 0.05 were considered significant.

Initially, the GSE243359 dataset obtained from the GEO repository underwent batch effect correction followed by data normalization (Figures 2A,B). Next, according to the predefined criteria, we identified DEGs in the GSE243359 dataset and intersected them with the CR gene list retrieved from the GeneCards database, resulting in 33 CR-DEGs (Figures 2C–E). Comprehensive somatic mutation profiling revealed extensive genomic alterations in HNSCC, with extracellular matrix-associated genes such as *LAMA3*, *TNC*, and *ITGB4* demonstrating the highest mutational burdens (Figure 2F). These results provide molecular clues of CR in HNSCC pathogenesis for HNSCC patients.

### 3.2 Identification of CR-associated shared DEGs in HNSCC patients

**FIGURE 3:**
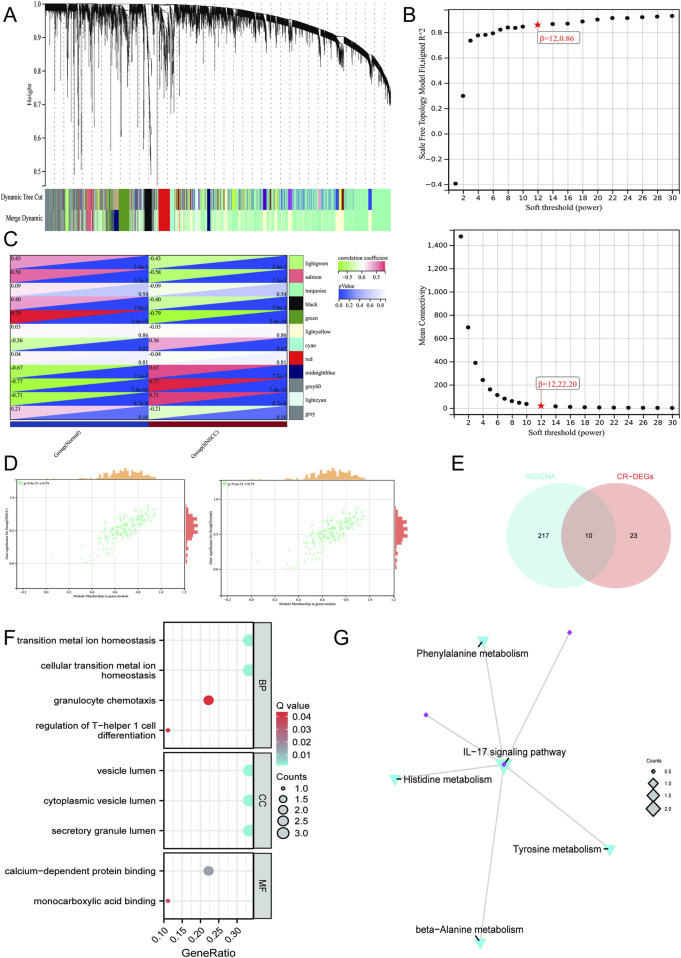
WGCNA for the identification of key CR-DEGs. **(A)** Gene co-expression modules with different colors under the gene tree. **(B)** β = 12 is chosen as the soft threshold based on the scale independence and average connectivity. **(C)** Association of gene co-expression modules with HNSCC and paired adjacent tissues. **(D)** Correlation analysis between gene significance and module membership in the green module. **(E)** Venn diagram of green module and CR-DEGs. **(F)** GO enrichment analysis of DEGs. **(G)** KEGG enrichment analysis of DEGs. Data with a threshold of p < 0.05 were considered significant.

Subsequently, a scale-free gene co-expression framework was established through WGCNA to pinpoint modules showing the strongest association with HNSCC. Guided by the criteria of scale independence and average connectivity, the soft-threshold parameter was optimized and set to β = 12 (Figures 3A,B). Correlation assessment between module eigengenes and clinical characteristics demonstrated that the green module (correlation coefficient = 0.79 and −0.79 and p-value = 2.4e-10) showed a markedly positive relationship with HNSCC progression (Figure 3C). Through WGCNA, we observed a pronounced positive association between the membership of genes in the green module and phenotypic relevance, suggesting that the genes clustered in this module serve as key regulators in the initiation and development of HNSCC (Figure 3D). Subsequently, we intersected the CR-DEGs with the green module and identified 10 shared candidate genes (Figure 3E). Integrated GO and KEGG enrichment analyses revealed that the candidate DEGs are critically involved in key biological processes such as maintenance of metal-ion homeostasis, granulocyte-mediated immune regulation, and vesicle trafficking and are significantly enriched in the IL-17 signaling pathway and multiple amino-acid metabolic cascades (Figures 3F,G). These results showed that highly associated CR-related shared DEGs were mainly involved in immune and metabolic reprogramming during HNSCC progression.

### 3.3 CR-associated optimal prognostic model construction and targeted gene enrichment

**FIGURE 4:**
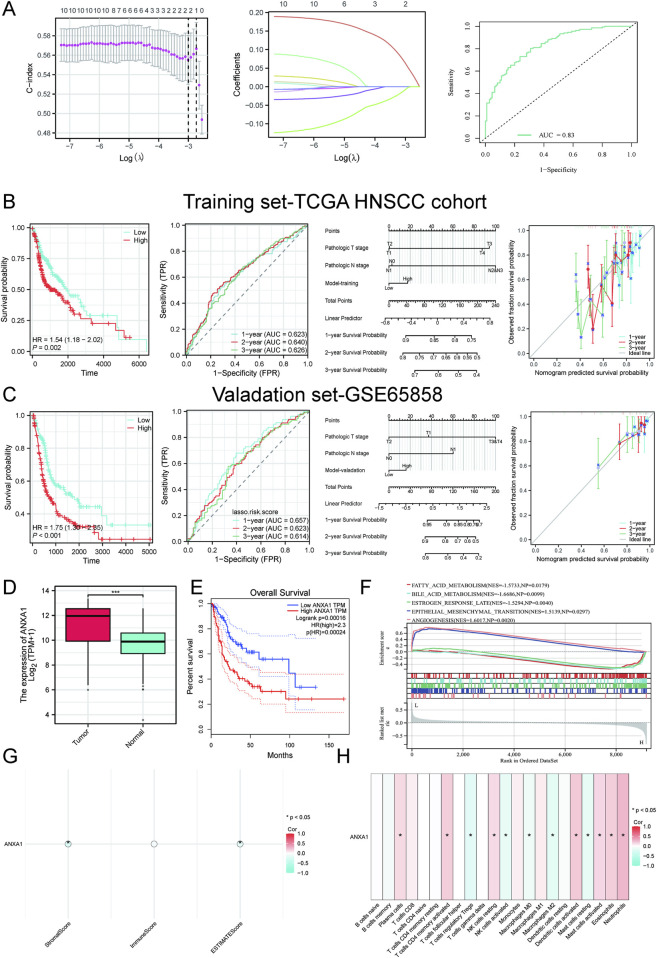
Machine learning framework for the construction of the CR-associated prognostic model. **(A)** LASSO regression results. **(B)** CR-associated prognostic model construction using TCGA-HNSCC cohort. **(C)** CR-associated prognostic model construction using the GSE65858 cohort. **(D)** Differential expression of ANXA1 between tumors and normal tissues. **(E)** KM curves linking ANXA1 between tumors and normal tissues. **(F)** Single-cell GSEA of ANXA1 in HNSCC patients. **(G)** CIBERSORT analysis of ANXA1 for HNSCC patients. **(H)** Correlation analysis between ANXA1 expression and the relative abundance of CIBERSORT-inferred immune cell types in HNSCC. Data with a threshold of p < 0.05 were considered significant.

By integrating LASSO–Cox regression and the TCGA-HNSCC cohort, we constructed a CR-associated prognostic model (Figure 4A). The ROC curve indicated that the CR-associated model achieved satisfactory efficacy and accuracy (Figure 4A). A prognostic risk model developed through LASSO successfully classified HNSCC patients into low- and high-risk categories across both TCGA-HNSCC and GSE65858 cohorts, exhibiting strong predictive accuracy, as reflected by the favorable prognostic efficacy (Figures 4B,C). Next, LASSO–Cox regression also confirmed a CR-associated hub gene, named *ANXA1*, which showed higher expression in HNSCC patients than in normal controls (Figure 4D). ANXA1 was also significantly associated with poorer overall survival of HNSCC patients (Figure 4E). Furthermore, the relationship between ANXA1 and immune and stromal heterogeneity was assessed (Figures 3F–H). Taken together, these results highlighted the cariogenic role of ANXA1 in HNSCC progression.

### 3.4 ANXA1 expression at the single-cell level in HNSCC patients

**FIGURE 5:**
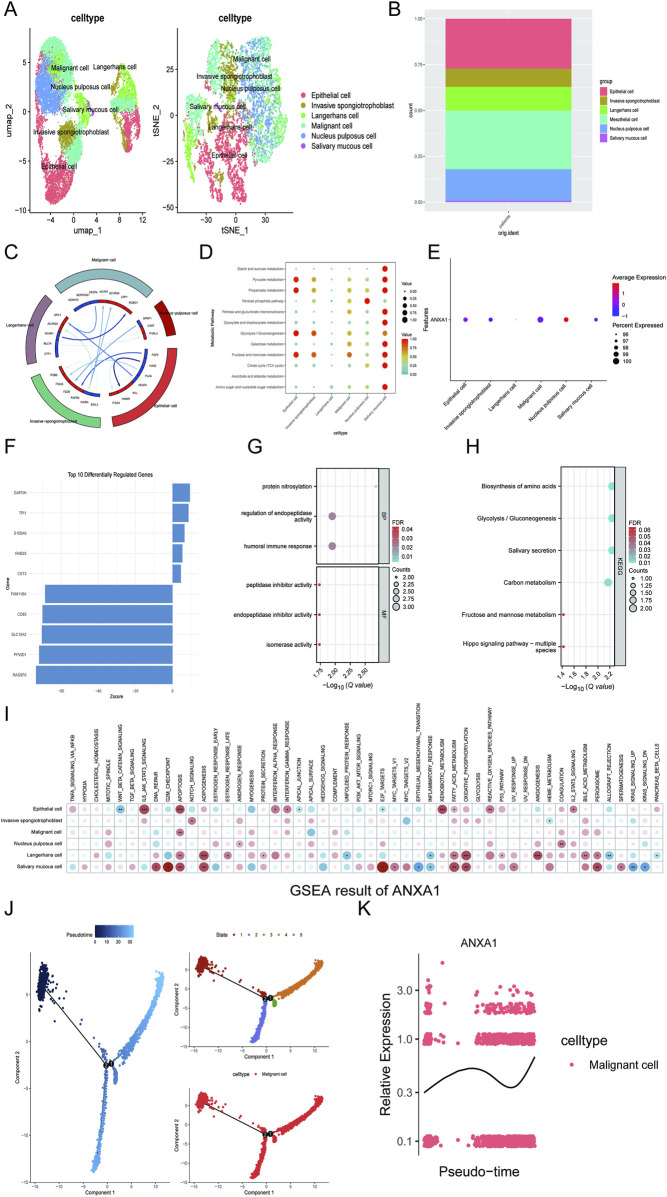
ANXA1 expression at the single-cell level in HNSCC patients. **(A,B)** Cell types in HNSCC patients. **(C)** Celltalker analysis. **(D)** Energy metabolism analysis in HNSCC patients. **(E)** Distribution of ANXA1. **(F–H)** KO of ANXA1 in malignant cells via AI-driven virtual cells. **(I)** Single-cell GSEA of ANXA1. **(J,K)** Monocle2 analysis. Data with a threshold of p < 0.05 were considered significant.

The scRNA-seq data utilized in this investigation were sourced from the GSE163872 dataset. Initially, the data underwent normalization and quality control (QC) procedures (Supplementary Figures S1A–C). To enhance the characterization of cell types, we categorized the cells into 17 distinct clusters based on the expression of established markers (Supplementary Figures S1D–F). Following annotation and inferCNV analysis, we confirmed the presence of six unique cell types: epithelial cells, invasive spongiotrophoblast cells, Langerhans cells, malignant cells, nucleus pulposus cells, and salivary mucous cells (Figures 5A,B). In addition, energy metabolism patterns and cell chat patterns (particularly malignant cells) among these six cell types were estimated (Figures 5C,D). We first discovered that ANXA1 was mainly distributed in malignant cells (Figure 5E). Monocle2 analysis revealed that there are two differentiation trajectories of malignant cells in HNSCC patients, and we also assessed the expression patterns of ANXA1 in malignant cells at temporal and spatial levels (Figures 5J,K). Furthermore, we performed AI-driven KO of ANXA1 in malignant cells and illustrated the top genes with altered expression with the corresponding interaction and functional patterns (Figures 5F–H). The results indicated that KO of ANXA1 mainly perturbed molecules that were highly associated with CR, such as T-helper differentiation and IL-17 pathway (Figures 5F–H). In addition, we also performed single-cell (sc) GSEA for gaining insights into the molecular and biological functions of ANXA1 among the six cell types (Figure 5I). Single-cell analysis results indicated that the crystal ecosystem of HNSCC patients highlighted the CR potential of ANXA1 in HNSCC patients.

### 3.5 Design of drug repurposing strategies for HNSCC patients and *in vitro* examination of ANXA1 in HNSCC

**FIGURE 6:**
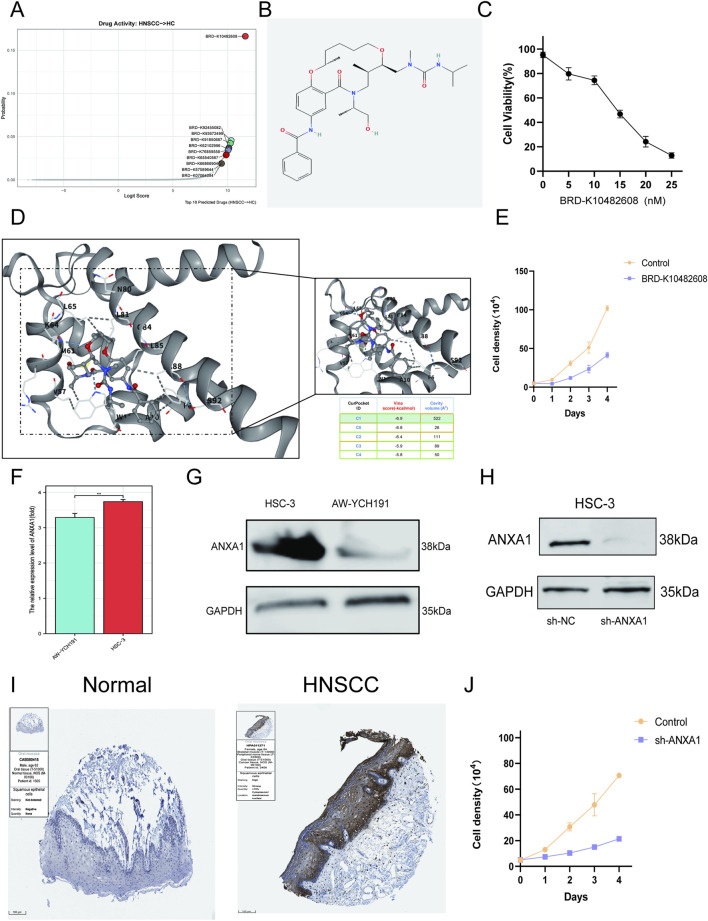
Prediction of the drug-targeting hub gene for the treatment of HNSCC. **(A)** AI-empowered drug screening for HNSCC treatment to bind ANXA1. **(B)** Two-dimensional structure of BRD-K10482608. **(C)** IC_50_ estimation. **(D)** Molecular docking model. **(E)** CCK-8 assays for estimation of the therapeutic efficacy of BRD-K10482608. **(F)** qRT-PCR estimation of ANXA1 expression in HSC-3 cell lines compared to that in normal control. **(G)** WB estimation of ANXA1 expression in HSC-3 cell lines compared to that in normal control. **(H)** WB examination of the knockdown efficacy of ANXA1 in HSC-3 cell lines. **(I)** Histological examination of ANXA1 in HNSCC tissue samples compared to that in normal samples. **(J)** CCK-8 assays for the estimation of ANXA1’s tumor-facilitating capacity. Data are presented as mean ± SD; *p < 0.05, **p < 0.01, and ***p < 0.001.

We performed AI-empowered drug screening (DrugReflector) in TCGA-HNSCC cohort for the identification of top 10 therapeutic agents targeting ANXA1 for the treatment of HNSCC (Figure 6A). We discovered that BRD-K10482608 can be considered an optimal one (Figure 6B). To estimate whether ANXA1 was the target for BRD-K10482608 treatment, we performed molecular docking. The docking analysis illustrated the stable binding cavity between ANXA1 and BRD-K10482608 (-6.9 kcal/mol), which illustrated that BRD-K10482608 may be considered a therapeutic agent for HNSCC treatment by targeting ANXA1 (Figure 6D). In addition, we examined the therapeutic efficacy of BRD-K10482608 in HSC-3 cell lines and discovered the anti-tumor potential of BRD-K10482608 against HNSCC, which showed cancer growth-restraining tendency (Figures 6C,E). We next estimated the expression level of ANXA1 in the HNSCC cell line compared to that in normal cell lines and observed that ANXA1 showed an upregulated expression trend at mRNA and WB levels (Figures 6F,G). In addition, we also restrained the expression of ANXA1 in the HSC-3 cell line via shRNA and discovered that inhibiting the expression of ANXA1 can restrain the growth of the HNSCC cancer cell line (Figures 6H,J). In addition, at the HNSCC histological level, ANXA1 showed upregulated expression patterns compared to those in normal controls (Figure 6I). These results provide concrete evidence for the cariogenic role of ANXA1 and the therapeutic potential of BRD-K10482608 in HNSCC.

## 4 Discussion and conclusion

HNSCC is a highly heterogeneous malignancy of the upper aerodigestive tract with steadily increasing global incidence and mortality. CR in HNSCC patients represents a major barrier leading to treatment failure, recurrence, and distant metastasis (Xu T. et al., 2023). By integrating bulk and single-cell transcriptomic data from TCGA and GEO databases with integrative bioinformatics analysis and AI pipelines, our study first identified the total landscape for CR in HNSCC at the molecular level. In addition, ANXA1 can be considered a potential predictive and therapeutic target indicative of HNSCC progression and CR pathogenesis. Furthermore, BRD-K10482608 can be considered a potential therapeutic agent targeting ANXA1 for the treatment of HNSCC.

CR is caused by multilayered mechanisms, primarily including ABC transporter-mediated drug efflux, extensive epigenetic remodeling that rewires transcriptional programs, and cellular plasticity (Mastrolonardo et al., 2025). In parallel, the tumor microenvironment, including dysregulation of CAFs, macrophages, and regulatory T cells, can promote immune evasion and hinder drug penetration (Um et al., 2025). In addition, HNSCC patients with CR face additional complications such as chronic obstructive pulmonary disease (COPD) and pulmonary fibrosis (Barnes et al., 2015; Shen et al., 2024). Hence, understanding CR mechanisms can provide insights into addressing the complications of HNSCC patients with CR, thus increasing their quality of life. In recent years, there has been significant advancement in the research targeting reverse CR in HNSCC patients. For example, PPARα-mediated lipid metabolism reprogramming can support anti-EGFR therapy resistance in HNSCC patients (Van den Bossche et al., 2025). The MRPL21–PARP1 axis can promote cisplatin resistance in HNSCC by inhibiting autophagy through the PI3K/AKT/mTOR signaling pathway (Guan et al., 2025). However, recent studies on chemoresistance in HNSCC have been limited by a focus on single mechanisms, and they do not offer a comprehensive CR atlas for HNSCC patients. Indeed, our study pointed out a multi-dimensional landscape for CR molecular mechanisms in HNSCC patients, which enhances their clinical translation. ANXA1, a calcium-dependent phospholipid-binding protein, functions as a critical molecular regulator of calcium homeostasis (Xiao et al., 2023). ANXA1 regulates membrane trafficking, cytoskeletal remodeling, inflammatory signaling, and immune modulation, and its aberrant overexpression has been linked to tumor invasion, metastasis, and therapy resistance across multiple cancer types (Li et al., 2022). In HNSCC, elevated ANXA1 expression promotes EMT and metabolic plasticity, enhances TGF-β/Wnt/β-catenin and KRAS signaling, and shapes an immunosuppressive microenvironment, thereby coupling diverse resistance mechanisms to aggressive clinical behavior and highlighting its dual value as both a prognostic marker and a druggable target (Xiao et al., 2023; Raulf et al., 2018; Garcia Pedrero et al., 2004). In addition, RPM2 can stabilize ANXA1 by activating the AKT signaling pathway, thereby regulating renal cancer sensitivity to sunitinib and PD-1 blockade (Xiong et al., 2021). Co-expression of ANXA1 and MHC-II also promotes PD-1/PD-L1 therapy resistance in breast cancer (Wang et al., 2025). However, the underlying mechanisms by which ANXA1 regulates resistance to HNSCC chemotherapy and immunotherapy have not been elucidated yet.

Overall, this is the first research study that discovered the global landscape of CR in HNSCC pathogenesis by constructing a CR-associated predictive and therapeutic model for HNSCC patients. However, it is crucial to recognize the limitations of our study. For example, the relatively small sample size limited the generalizability of our findings, thereby warranting further validation through larger clinical trials to confirm the relevance of our results in the treatment of HNSCC. Moreover, future pre-clinical and clinical studies are needed to elucidate the molecular mechanisms by which ANXA1 regulates CR and validate the therapeutic potential of BRD-K10482608 in HNSCC.

## Data Availability

The data analyzed in this study are publicly available. TCGA-HNSCC (TCGA-HNSC) bulk RNA-seq expression profiles and corresponding clinical information were obtained from the Genomic Data Commons (GDC) portal: https://portal.gdc.cancer.gov/projects/TCGA-HNSC. GEO datasets were downloaded from NCBI GEO under accession numbers GSE6631 (https://www.ncbi.nlm.nih.gov/geo/query/acc.cgi?acc=GSE6631), GSE65858 (https://www.ncbi.nlm.nih.gov/geo/query/acc.cgi?acc=GSE65858), and GSE163872 (https://www.ncbi.nlm.nih.gov/geo/query/acc.cgi?acc=GSE163872). Histological images were obtained from The Human Protein Atlas (https://www.proteinatlas.org/), and chemotherapy resistance–associated genes were retrieved from GeneCards (https://www.genecards.org/). No new datasets or code were generated in this study.
