# Supplementary figures and images for: Characterization of ANXA1 in chemotherapy resistance of head and neck squamous cell carcinoma: insights from artificial intelligence and integrative bioinformatics analysis
Source: Front Cell Dev Biol. 2026 Feb 26;14:1769105. doi: 10.3389/fcell.2026.1769105 (PMC12979454; doi:10.3389/fcell.2026.1769105)

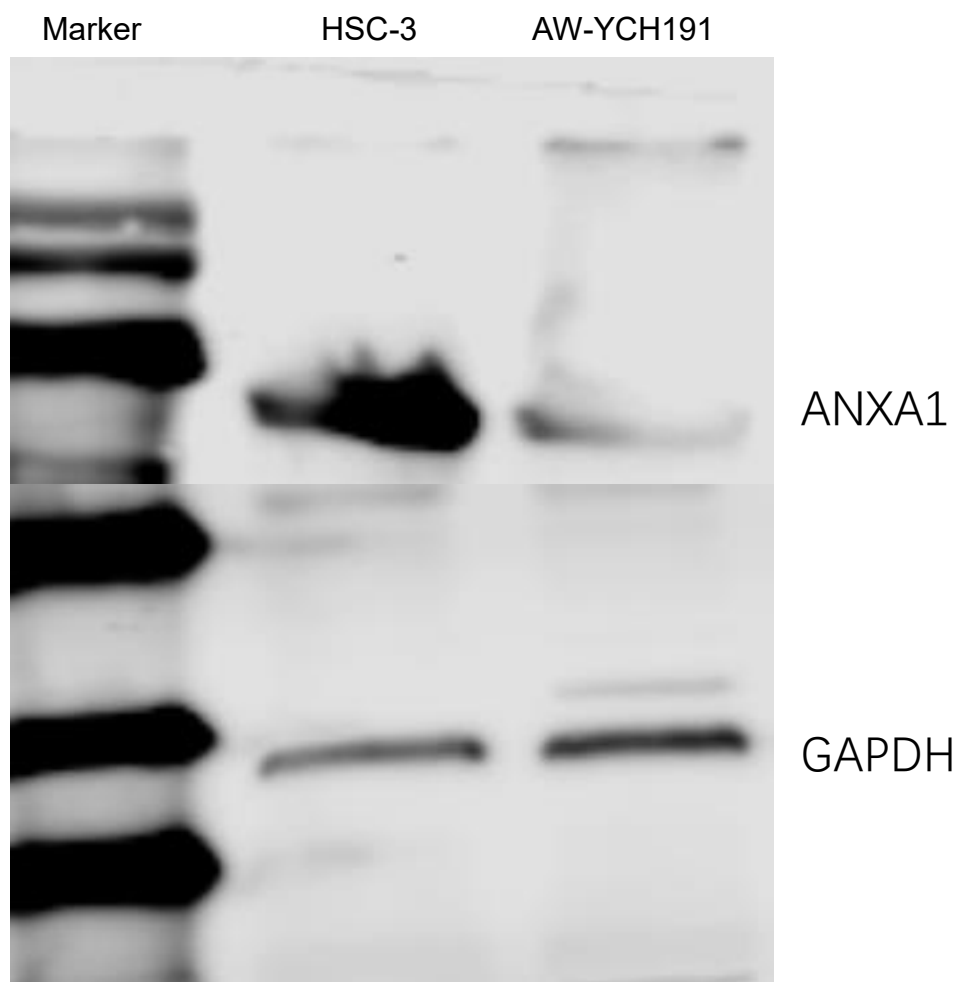

Supplement: Supplementary file 1 [file DataSheet1.pdf]
