# Supplementary material for: Characterization of ANXA1 in chemotherapy resistance of head and neck squamous cell carcinoma: insights from artificial intelligence and integrative bioinformatics analysis
Source: Front Cell Dev Biol. 2026 Feb 26;14:1769105. doi: 10.3389/fcell.2026.1769105 (PMC12979454; doi:10.3389/fcell.2026.1769105)
Supplement: Supplementary file 2 [file DataSheet2.docx]

**
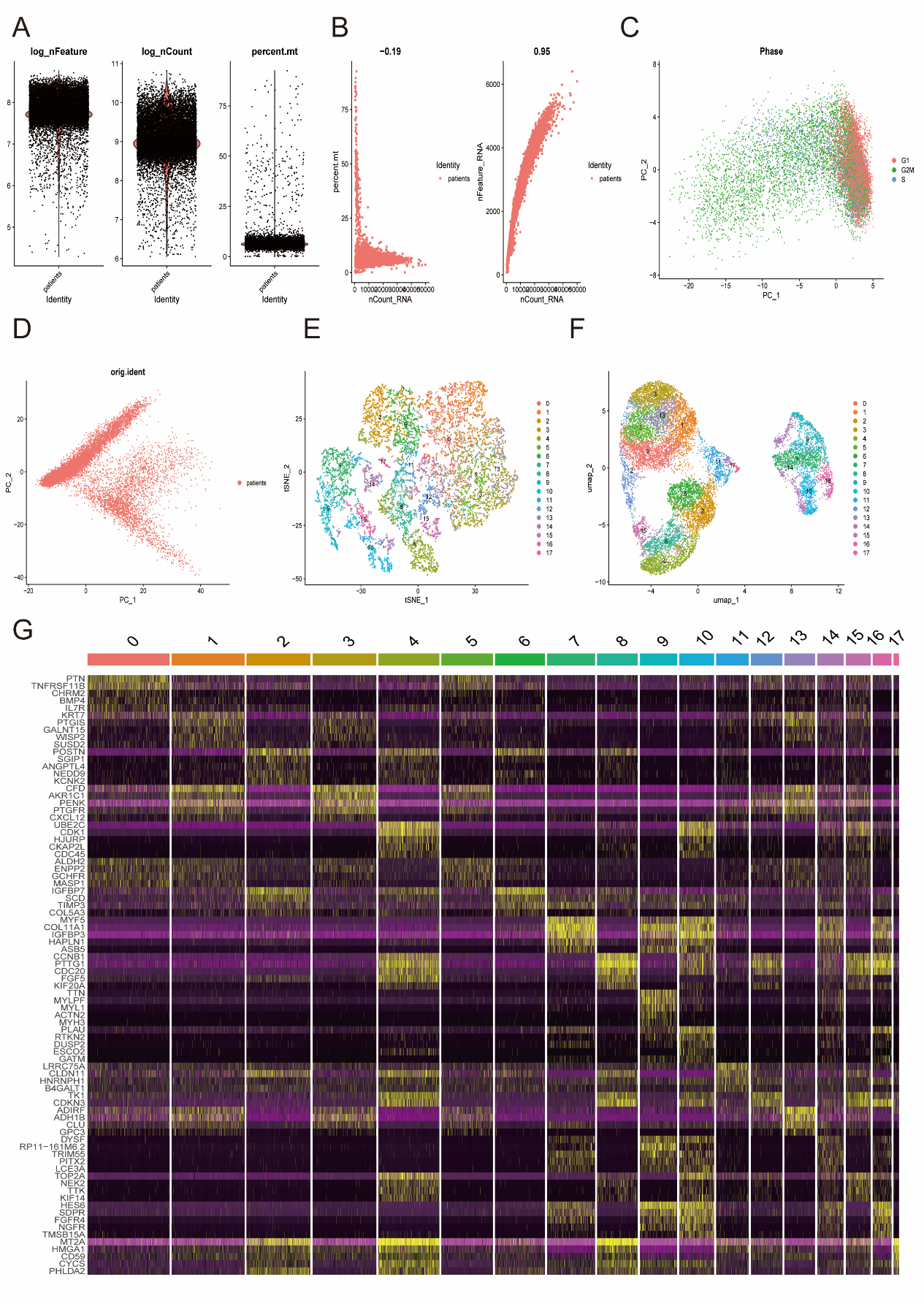
**

**Figure S1 Pre-processing of single-cell data. (A-C)** Quality control metrics of single-cell RNA-seq data. **(D)** Cell cycle scoring and distribution. **(E-F)** PCA and UMAP plots of cell clusters. **(G)** Heatmap of marker gene expression.
